# Acceptability of a Novel Parent‐Mediated Executive Function Intervention for Young Children With Down Syndrome in Italy

**DOI:** 10.1111/jar.70038

**Published:** 2025-03-19

**Authors:** Sara Colaianni, Madison M. Walsh, Sara Onnivello, Miranda E. Pinks, Chiara Marcolin, Kaylyn Van Deusen, Elisa Rossi, Nathaniel R. Riggs, Francesca Pulina, Lisa Daunhauer, Deborah J. Fidler, Silvia Lanfranchi

**Affiliations:** ^1^ Department of Developmental and Socialization Psychology University of Padua Padua Italy; ^2^ Department of Human Development & Family Studies Colorado State University Fort Collins Colorado USA

**Keywords:** Down syndrome (DS), executive function (EF), executive function intervention, intellectual disability (ID), intervention acceptability

## Abstract

**Background:**

People with Down syndrome (DS) are predisposed to challenges with executive functions (EF), which are crucial for adaptive outcomes and academic success. Early interventions targeting EF are therefore critical. The present study analysed Italian data on the acceptability, enjoyability and household implementation of EXPO (EXecutive function Play Opportunities), a novel caregiver‐mediated intervention designed to strengthen EF skills in young children with DS.

**Methods:**

Nineteen families of children aged 42–93 months participated. Caregivers completed questions via a smartphone app and provided feedback through Mid‐Point and Exit questionnaires.

**Results:**

Implementation and enjoyment remained consistent throughout the intervention. Caregivers reported positive effects of EXPO on children's everyday life skills and found the coaching sessions beneficial for successful program implementation.

**Conclusion:**

The EXPO intervention is acceptable and enjoyable for both caregivers and children, with caregivers reporting perceived improvements in children's everyday life skills after participating in the program.

**Summary**

- Consistent implementation and enjoyment of EXPO by families of children with DS.
- Acceptability of EXPO for families of children with DS.
- Effects of EXPO on everyday life skills of young children with DS.
- Implications for future efficacy trials.

## 1 Introduction

Executive functions (EF) are the cognitive regulatory skills necessary for planning and implementation of goal‐directed behaviours (Lee et al. 2015). Most models of EF include the component processes of working memory, inhibition, flexibility and planning (Diamond 2013), with some models including additional domains such as emotional control as well (Gioia et al. 2003). Individuals with Down syndrome (DS) frequently demonstrate EF difficulties (Tungate and Conners 2021), with a childhood EF profile characterised by relative strengths in emotional control and more pronounced difficulties with shifting, planning, and inhibition, and even greater difficulties with working memory (Onnivello et al. 2022).

The importance of EF for skill acquisition across various developmental domains, such as learning and daily living skills, has been widely highlighted in typical development (Benavides‐Nieto et al. 2017; Diamond 2013; Mazuka et al. 2009). These aspects have also been studied in individuals with DS. For example, Onnivello et al. (2022) demonstrated the significant influence of EF on various domains of daily life, particularly during school age. Working memory and inhibition appear to play crucial roles in the school setting, with laboratory performance on these dimensions predicting academic achievement in young children with DS (Will et al. 2017).

EF is also related to behavioural dysregulation in the form of inattention, rule‐breaking, aggression, and externalising behaviours in children with DS (Esbensen et al. 2021). In particular, inhibitory control predicts externalising behaviour challenges at home and at school in children with DS (Esbensen et al. 2021). Inhibition also predicts rule‐breaking behaviours at school, as well as aggression at both home and school (Esbensen et al. 2021). Recent work has also demonstrated that the ability to shift between tasks predicts rule‐breaking behaviour, aggression, and externalising behaviours at home, and emotional control predicts aggressive and externalising behaviours at home (Esbensen et al. 2021).

### 1.1 EF Intervention in DS

Since EF is crucial for successful planning and functional performances in many domains of daily life, targeting these cognitive processes may be a beneficial intervention strategy for individuals with DS, with the potential to initiate adaptive developmental cascades onto more advanced skill acquisition. The emergence of childhood EF vulnerabilities in DS suggests that intervention may be particularly beneficial before the transition to formal schooling in the primary grades. Improving these foundational skills in the early years of a child's life may positively influence school transitions, participation, functional skills, and later employment outcomes.

There are currently several barriers to EF intervention participation for young children with DS. Existing child‐focused EF interventions often require cognition and language skills that are more advanced than generally observed in preschool‐aged children with DS, making these interventions inaccessible for most young children with DS before the transition to Kindergarten (Bennett et al. 2013). Logistical factors also pose challenges for families of young children with DS. Travel to a specialised centre every week, and possibly more than once per week, can be difficult due to the numerous weekly activities in which families are involved. Implementing trainings at home can significantly reduce logistical burdens for families. Previous studies have demonstrated the possibility of parent‐mediated intervention to be implemented at home under the supervision of an expert to improve aspects of cognitive functioning in children and adolescents with DS (e.g., Lanfranchi et al. 2021; Pulina et al. 2015). Other working memory training studies in DS conducted in a home‐based setting have also demonstrated their efficacy in improving the targeted domain (Conners et al. 2008; Pulina et al. 2015). Additionally, computerised numeracy training has been shown to be effective when administered at home by parents (Lanfranchi et al. 2021). Therefore, given these findings, a parent‐implemented approach to EF intervention in DS may increase accessibility and potential efficacy.

### 1.2 EXecutive Function Play Opportunities: EXPO

To address current barriers to EF intervention participation for young children with DS, the ‘EXPO (EXecutive function Play Opportunities)’ intervention was developed. EXPO is a caregiver‐mediated EF intervention for preschoolers with DS, designed and refined through the international collaboration of two research groups, at Colorado State University and University of Padova. The community‐engaged, iterative intervention development and refinement process is described elsewhere in detail (Lanfranchi et al. 2024; Walsh et al. 2024).

The current structure of EXPO (version 2.0) has a duration of 12 weeks and involves play‐based activities for caregivers and their child with DS designed to strengthen EF. The program focuses on building various aspects of EF and on consolidating skills. The 12 weeks are divided into 6 activity blocks, each lasting 2 weeks. The first block (weeks 1 and 2) is dedicated to interactional *Foundations*, aiming to strengthening behaviours such as eye‐contact, request and choice making, imitation of actions, and turn taking. These skills are essential for carrying out the activities planned for the subsequent weeks of the EXPO intervention. From the second to the fifth block, the activities target specific EFs: *Working Memory* (block 2; weeks 3 and 4), *Inhibition* (block 3; weeks 5 and 6), *Flexibility* (block 4; weeks 7 and 8), and *Planning* (block 5; weeks 9 and 10). The final block (weeks 11 and 12) focuses on the *Consolidation* of EF abilities acquired during the previous weeks of intervention. The activities are either adaptive, based on routine actions of daily life, or play‐based, in the form of games that families can play together. Activities are carried out by caregivers at home, incorporating activities within daily routines and other members of the household that are present (e.g., siblings, grandparents). After enrolling in EXPO, caregivers receive materials that include a comprehensive guide explaining the intervention rationale, activity structuring suggestions, and activity directions. Caregivers also have access to an EXPO website with digital versions of materials. Caregivers are supported across the 12‐week intervention by an EXPO coach who has experience in early intervention. Weekly coaching sessions last about 30 min and are structured to reflect on activities carried out in the previous week, to provide an opportunity for caregivers to ask questions about EXPO activities, and to choose and plan activities for the next week.

In preparation for a larger‐scale trial, a preliminary implementation of EXPO 2.0 was conducted in Italy and the US to evaluate its feasibility, acceptability, and preliminary efficacy (see Pinks et al. forthcoming; Walsh et al. forthcoming). Preliminary efficacy based on the pilot implementation is characterised in depth elsewhere (see Pinks et al. forthcoming). However, due to the parent‐mediated nature of the intervention, an additional in‐depth evaluation of intervention acceptability is warranted, and details regarding the nature of the implementation of EXPO activities across households can shed light on the various ways that families incorporated intervention activities into their daily lives. Assessing acceptability is crucial for determining how suitable, satisfying, and appropriate clinical or educational treatments are for families. It helps to establish the enjoyability and ease of implementation while identifying both positive and negative aspects to refine and improve the intervention's procedures (Stojanovik et al. 2023; Stone‐Heaberlin et al. 2024). Furthermore, although Italian and US families may demonstrate similarity along various sociodemographic dimensions, the cultural, linguistic, and national context of implementation are critical to consider when evaluating intervention acceptability. For this reason, the present study focused specifically on analysing data collected from an Italian sample. The objective was to assess the acceptability and enjoyability of EXPO 2.0 among caregivers of young children with DS in Italy and to examine the details of its implementation in household settings. Acceptability and enjoyability for the USA sample are described elsewhere (see Walsh et al. forthcoming). Data from activity logs were analysed to describe the time, place, and other contextual factors regarding EXPO activity sessions. Intervention acceptability was evaluated by analysing enjoyability ratings entered after each activity session, and through caregiver feedback evaluations provided via caregiver surveys at intervention Mid‐Point (MP) and Exit (ET) (note: preliminary feasibility and efficacy data of the EXPO 2.0 pilot implementation trial are reported elsewhere [Pinks et al. forthcoming]). Finally, parents' perception of the intervention, as a further measure of acceptability, was assessed. Results will inform further revision and refinement of EXPO in preparation for a larger‐scale trial.

## 2 Methods

### 2.1 Participants

Participants were children with DS and their caregivers located in two regions in Italy, recruited through parent advocacy groups. Of the initial 20 children who enrolled in the study with their families, 10 were males and 10 were females. Children were between the ages of 42 and 93 months old (*M* = 65.9; SD = 15.12). One family withdrew from the study due to unexpected logistical challenges. The characteristics of the sample are described in Table 1.

**TABLE 1:** Characteristics of the sample.

	*M*	SD	Min‐max
Chronological age (months)	65.9	15.12	42–93
Mental age^a^ (months)	26.85	8.1	5–38

^a^ Mental age derived from the Griffiths Scales of Child Development, Third Edition (Lanfranchi et al. 2019).

### 2.2 Procedure

Caregivers gave verbal and written consent before beginning their participation in EXPO. Baseline assessments were conducted at the research laboratory or at community spaces that were geographically convenient for families. Baseline and post‐intervention assessments included direct and proxy report measures of development, adaptive behaviour, EF, and parent–child relationship (see Pinks et al. forthcoming, for details regarding intervention outcome measures).

Upon enrolment, caregivers were assigned an EXPO coach who was a study team member with clinical and/or early intervention expertise. After the baseline assessment, a more specific introductory individual coaching meeting was completed before the start of the intervention. Weekly individual coaching sessions allowed caregivers to discuss the activities carried out in the previous week, ask questions about the program, and plan activities for the following week. After completing the 12‐week intervention, families completed a post‐intervention assessment with the same procedures as the baseline evaluation. Caregivers also completed an anonymous online feedback questionnaire at Mid‐Point (MP; 6th week of the program) and Exit (ET; 12th week of program) to provide feedback on the enjoyability of activities and their perceptions of the EXPO program.

### 2.3 Measures

#### 2.3.1 Quantitative Caregiver Ratings of EXPO via Activity Log

Caregivers were instructed to complete a brief ‘EXPO Activity Log’ entry via smartphone application upon completion of each home‐based activity session. To better understand the household implementations of EXPO, activity log prompts included questions regarding who led the activity with the child, what time of day it was completed, the approximate duration of the activity, and where it was completed. Log entries also included questions regarding caregiver and child activity enjoyment. Enjoyability levels for both child and caregiver were rated on a 5‐point Likert‐type scale.

#### 2.3.2 Qualitative Caregiver Evaluation of EXPO via Feedback Questionnaires

An anonymous caregiver feedback questionnaire was administered online using the Qualtrics platform (Qualtrics, Provo, UT) at intervention MP and ET. At both time points, caregivers were asked to provide overall appraisals of EXPO activities, perceived positive aspects of EXPO, and perceived challenging aspects of EXPO, their preferences for types of activities offered within EXPO (e.g., play‐based vs. adaptive), as well as the more liked and disliked activities. Further space was given for additional comments. At ET, caregivers were also asked about their usage of the EXPO App and the EXPO platform, the usefulness of the coaching sessions, the perceived effects of EXPO on their child's development and daily life, and their perception of child skill acquisition. At the end of the questionnaire, caregivers had the opportunity to provide open‐ended suggestions for improving the program. Open responses were registered and categorised by theme by two separate evaluators (100% of concordance), then the frequency of each response was calculated. Questionnaires are available in Appendices A and B.

## 3 Results

### 3.1 Quantitative Evaluation of EXPO

#### 3.1.1 Household Implementation of EXPO Activities

Italian households recorded a total of 1949 activity sessions. The majority of sessions were led by mothers (80.9% of sessions), but sessions were also led by fathers (11.1% of sessions), siblings (6.9% of sessions), babysitters (0.5% of sessions), grandparents (0.3% of sessions) and other caregivers (0.3% of sessions). Many activities were conducted in the afternoons (54% of sessions), although some activity sessions took place in the evening (25.7%) or in the morning (20.3%). Most activity sessions were completed in living rooms (62.6% of sessions), and sessions were also conducted in bedrooms (11.7% of sessions), kitchens (11.5% of sessions), bathrooms (7.3% of sessions) and other miscellaneous locations (6.8% of sessions).

Caregivers reported that 18.8% of activities were implemented during family routine moments (such as dressing, mealtime, or bedtime), while 81.2% of activities were carried out during specific times designated for EXPO. In the majority of activity sessions (79.4%), the caregiver and child with DS participated in the activity one‐on‐one. In 20.6% of sessions, other people participated in the activities as well. Out of 391 sessions in which other family members joined in the EXPO activities, 57.5% included siblings, 26.8% included the other caregiver, 6.1% included the whole family, and 4.9% included grandparents. Therapists also participated in the activities in 1.8% of sessions, other relatives participated in 13% of sessions, cousins participated in 1% or sessions, and the mother's partner took part in 0.6% of sessions.

EXPO activities were designed to be completed in 5–15 min. Caregivers reported that half of the activity sessions (51.1%) were completed in 5–10 min, 33.2% of sessions were completed in 5 min or less, 12.5% of sessions were completed in 11–15 min, and 3.2% of sessions were completed in more than 15 min. Out of 1930 activity sessions completed by caregivers, in 96.3% of them, caregivers reported that nothing interfered with the implementation of the activity. Only 3.7% reported that something influenced the course of the session, such as child fatigue or illness, or environmental distractions (e.g., siblings interrupting sessions, noises).

Implementation was mostly consistent across the 12 weeks of the intervention, with no notable decline in participation/implementation. Somewhat fewer activities were implemented during the Flexibility (*M* = 15.9, weeks 7 and 8) and the Consolidation (*M* = 15.3, weeks 11 and 12) blocks. Table 2 reports the mean number of activities implemented for each activity block.

**TABLE 2:** Mean number of activities (*M*) implemented for each activity block.

	Foundations weeks 1–2	Working memory weeks 3–4	Inhibition weeks 5–6	Flexibility weeks 7–8	Planning weeks 9–10	Consolidation weeks 11–12
*M*	17.9	16.7	17.2	15.9	19.8	15.3
SD	10.7	8.4	8.9	8.1	8.5	8.5
Min	3	0	2	3	7	3
Max	40	33	40	33	33	37

*Note:* Each activity block lasts 2 weeks.

#### 3.1.2 Activity Enjoyment Logs

The level of child and parental enjoyment was reported by caregivers using a 5‐point Likert‐type scale: 1 = ‘really did not enjoy’; 2 = ‘did not enjoy’; 3 = ‘neutral’; 4 = ‘enjoyed’; 5 = ‘really enjoyed’. Table 3 reports the frequencies reached for each level of enjoyment. Caregivers reported that children enjoyed the majority of activities. Out of a total of 1929 activity sessions, caregivers reported that children ‘really enjoyed’ 32.3% of sessions and ‘enjoyed’ 56.6% of sessions. Caregivers reported children had a neutral attitude in 9.8% of sessions, ‘did not enjoy’ 1.3% of activity sessions and ‘really did not enjoy’ 0.1% of activity sessions. Caregivers reported similar levels of enjoyment for themselves, rating 26.7% of sessions as ‘really enjoyed’, 65.2% of activity sessions as ‘enjoyed’, 7.3% of sessions as ‘neutral’, and only 0.8% of activity sessions as ‘did not enjoy’. Enjoyment remained consistent across the 12 weeks of the intervention, with no notable decline in caregiver or child enjoyment. Both caregiver and child enjoyment were highest during the last two blocks of the intervention. Table 4 reports the mean and standard deviation values of child and caregiver enjoyment. Enjoyment was consistent through all the blocks.

**TABLE 3:** Frequency of child and caregivers enjoyment for each activity block.

		Really enjoyed (%)	Enjoyed (%)	Neutral (%)	Did not enjoy (%)	Really did not enjoy (%)
Foundations	Child	46.5	41.2	11.2	1.1	0.0
	Caregiver	33.1	57.1	9	0.8	0.0
Working memory	Child	28.6	51.2	17.5	2.7	0.0
	Caregiver	23.5	63.9	11.1	1.5	0.0
Inhibition	Child	27.1	61.9	9.8	0.9	0.3
	Caregiver	21.7	70.2	7.1	0.9	0.0
Flexibility	Child	26.9	61.9	9.1	2.1	0.0
	Caregiver	24.8	65.7	8.7	0.7	0.0
Planning	Child	32.7	61.7	5.0	0.6	0.0
	Caregiver	28.9	67.8	2.9	0.3	0.0
Consolidation	Child	29.0	64.5	5.8	0.7	0.0
	Caregiver	27.3	67.6	4.4	0.7	0.0
TOT	Child	32.2	56.6	9.8	1.3	0.1
	Caregiver	26.7	65.2	7.3	0.8	0.0

**TABLE 4:** Means of child and caregivers enjoyment expressed on a 5‐point Likert‐type scale for each block.

	Foundations	Working memory	Inhibition	Flexibility	Planning	Consolidation
Child enjoyment (*M*)	4.22	4.08	4.13	4.12	4.34	4.27
Child enjoyment (SD)	0.41	0.16	0.24	0.33	0.33	0.32
Caregiver enjoyment (*M*)	4.18	4.03	4.13	4.12	4.31	4.28
Caregiver enjoyment (SD)	0.15	0.17	0.19	0.24	0.32	0.37

### 3.2 Qualitative Evaluation of EXPO

#### 3.2.1 Caregiver Perceptions Regarding Intervention Experience

**FIGURE 1:**
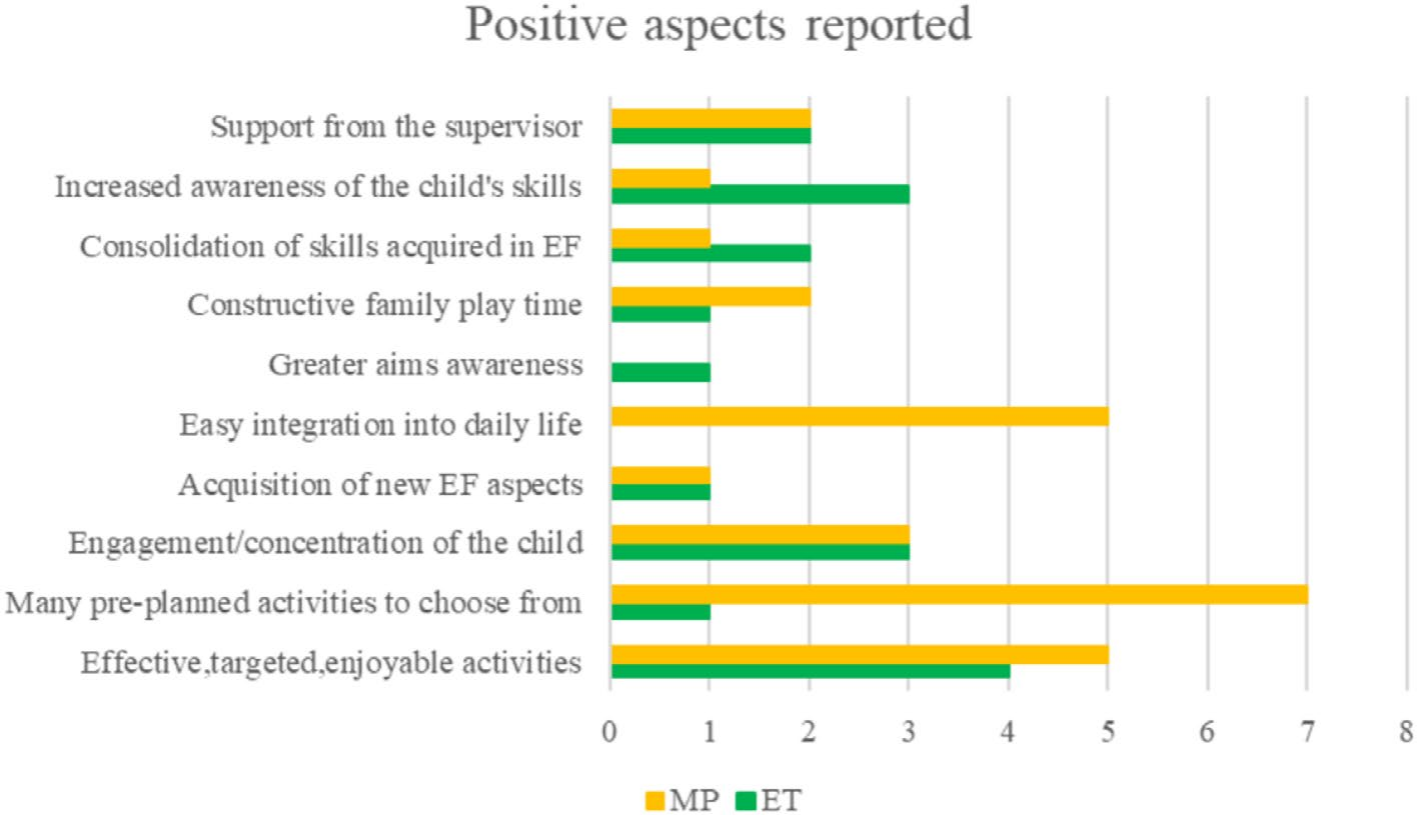
Frequency of positive aspects of EXPO expressed by families at MP and ET.

**FIGURE 2:**
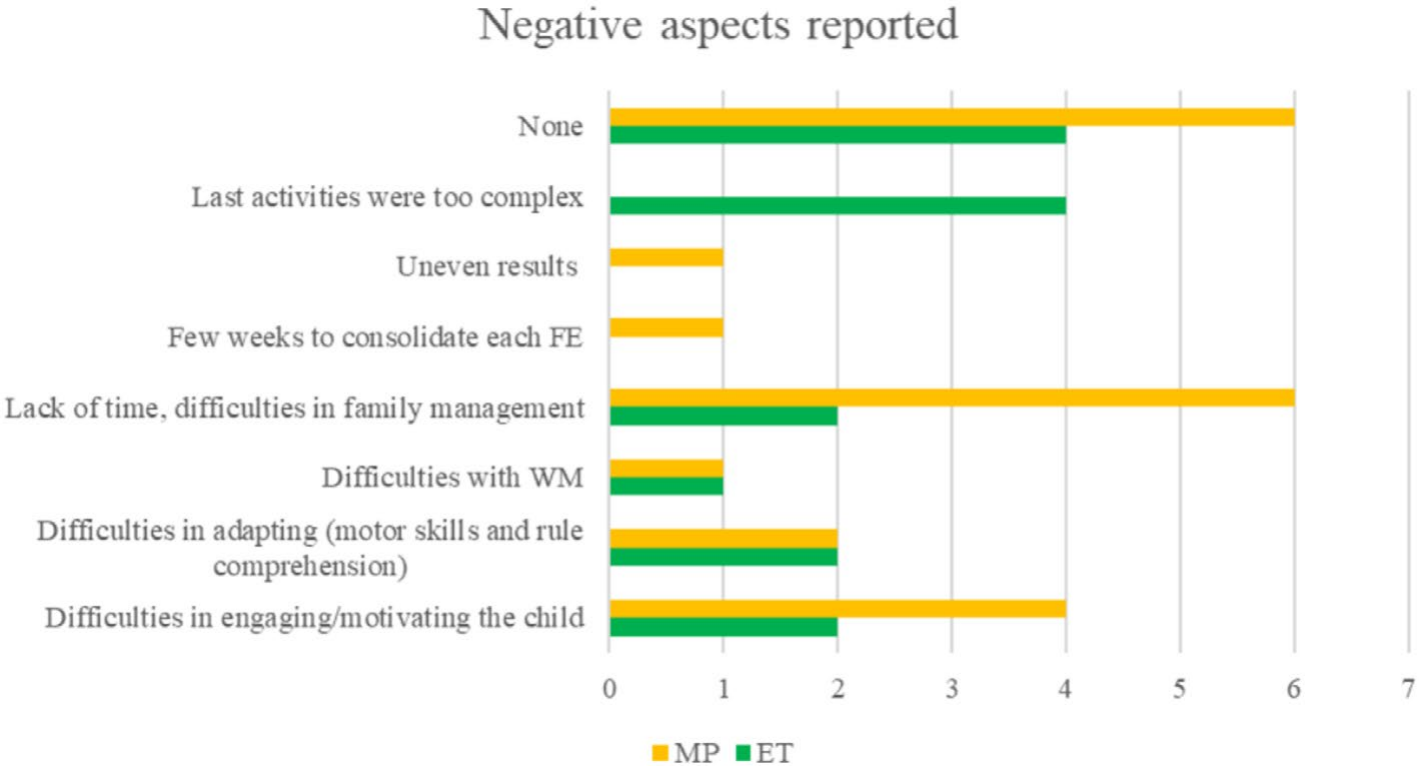
Frequency of negative aspects of EXPO expressed by families at MP and ET.

**FIGURE 3:**
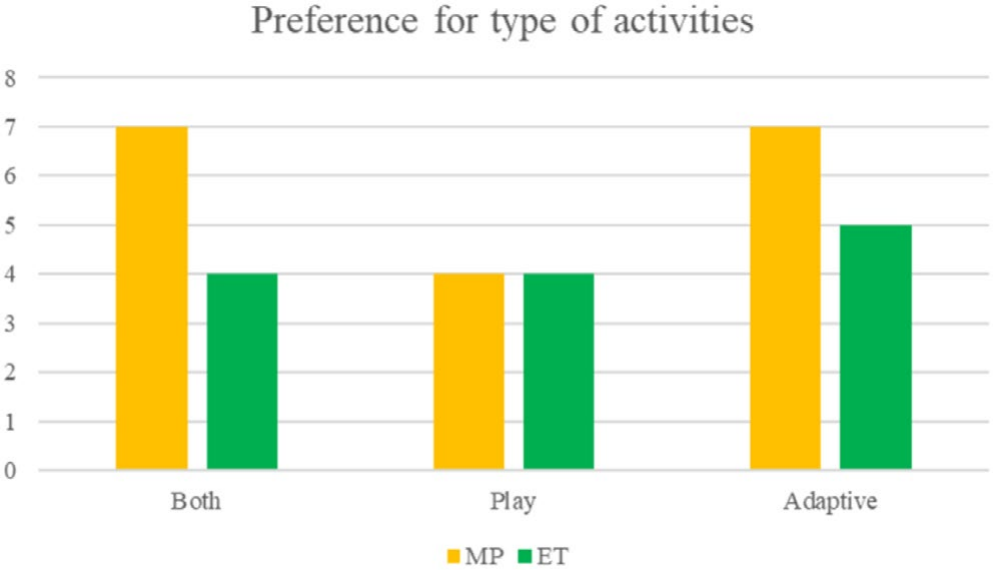
Frequency of preferences for types of activities at MP and ET.

Eighteen families completed the MP feedback questionnaire, and 13 families completed the ET feedback questionnaire. Frequencies of MP and ET responses are reported in Figures 1, 2, 3.

##### 3.2.1.1 Positive Aspects of EXPO

All families who completed the feedback MP and ET questionnaires were satisfied with EXPO and described several positive aspects of the program (Figure 1). In response to prompts regarding positive aspects of EXPO, nine families appreciated the EXPO materials, describing the activities as effective, targeted, and enjoyable. Additionally, eight families valued the variety of pre‐planned activities to choose from. Six families reported positive engagement and focused attention by the child, and five families suggested that the program offers activities that easily integrate into daily life. Four families highlighted the importance of receiving support from the coach, and another four families noticed an increased awareness of the child's skills. Three families observed a consolidation of skills learned by the child in EF, while two families noted the child's acquisition of new EF skills. Three families reported more constructive playtime together, and one family reported the child's greater awareness of the goals.

##### 3.2.1.2 Negative Aspects of EXPO

In response to prompts regarding perceived negative aspects of EXPO (see Figure 2), eight families reported the lack of time to implement the activities and struggling with family management; six families had some difficulties in engaging or motivating their child to participate; and four families reported some difficulties in adapting the activities for their child's motor and comprehension skills. Additionally, two families observed their child's difficulties with WM during activities, which made them more aware of this specific area of challenge. One family expressed concern that 2 weeks might be too short a period to consolidate the EF skills and mentioned needing more time to organise and adapt activities to fit into their daily routine and commitments. Another family noticed uneven results across the different EF, as they had expected a uniform enhancement of each skill. Four families reported difficulties with implementing the activities from the last two blocks, finding them too challenging for their children to understand and engage with. Ten families did not identify any negative aspect of EXPO.

##### 3.2.1.3 Preference for Type of Activities

The EXPO intervention offers options for adaptive activities (e.g., everyday actions, and tasks of daily living) and play‐based activities (e.g., games). Families reported that they appreciated both types of activities (Figure 3). Twelve families preferred adaptive activities (MP *n* = 7; ET *n* = 5); 8 families preferred play‐based activities (MP *n* = 4; ET *n* = 4); and 11 families liked both adaptive and play‐based activities (MP *n* = 7; ET *n* = 4).

##### 3.2.1.4 Caregiver Perceptions of Effects of EXPO on Daily Life

The ET feedback questionnaire also asked caregivers about perceived effects of EXPO on their children's daily lives, which was considered to be a further measure of the intervention's acceptability among families. It is important to note that answering this question was optional, and it was an open‐ended question. Of the 13 respondents, 12 reported observing at least one positive effect (Table 5). In response to open‐ended prompts, caregivers perceived that their children demonstrated more daily independence (*n* = 3); more cooperation in daily activities (*n* = 2); more structured individual play (*n* = 1); more constructive playtime together (*n* = 2); better reciprocity and turn‐taking (*n* = 1); more attention and concentration (*n* = 3); better problem solving (*n* = 1); more maturity (*n* = 1); and less frustration (*n* = 1). Five caregivers also reported perceiving that other adults who work with their children, including therapists, teachers, and babysitters, noticed positive effects following the children's participation in EXPO (Table 5).

##### 3.2.1.5 Perceived Utility of Coaching

All 13 families that decided to answer this optional open‐ended question expressed positive feedback regarding the weekly coaching sessions (Table 5), reporting that they provided opportunities for caregivers to discuss activities that were previously carried out (*n* = 6) and choose and plan the next activities (*n* = 4). Additionally, caregivers reported that coaches provided a helpful explanation of the activity instructions (*n* = 4) and discussed modifications to tailor the activities to their child (*n* = 4). Caregivers reported that coaches provided effective strategies to achieve goals (*n* = 5), sustained their motivation (*n* = 2) and offered support (*n* = 1).

**TABLE 5:** Number of families who provide responses on (1) EXPO effects on children's daily life, (2) other people noticing EXPO effects and (3) aspects in which coaching was useful.

Effects on daily life	*n*	Others that reported EXPO effects	*n*	Utility of coaching	*n*
Autonomies	3	Educational figures	2	Discussion regarding activities	6
More attention/concentration	3	Therapists	1	Useful strategies to achieve goals	5
Cooperation in daily activities	2	Teachers	1	Better explanation of activities	4
Constructive playtime together	2	Baby‐sitter	1	Activity adaptation	4
Reciprocity/turn taking	1			Choice and planning of activities	4
Less frustration	1			Caregiver motivation	2
More structured individual play	1			Support for extra EXPO aspects	1
More problem solving	1				
More maturity	1				

##### 3.2.1.6 How Can EXPO Be Improved?

Caregivers also provided feedback regarding how best to improve EXPO. One family suggested providing more opportunities to consolidate acquired skills (*n* = 1). Other suggestions were increasing the number of activities related to everyday life (*n* = 1) and raising the initial level of difficulty of the activities (*n* = 1). One family recommended introducing activities with low motor involvement (*n* = 1), while another expressed the need for individual activity adaptation (*n* = 1). Finally, one family felt the need for moments of sharing with other caregivers involved in EXPO (*n* = 1). Seven families did not answer this question (*n* = 7).

##### 3.2.1.7 General Comments

Parents were given the opportunity to provide general comments at the end of each questionnaire. Several parents were grateful for the opportunity to participate in this experience (*n* = 8), described EXPO as easy, enjoyable, useful, and well‐structured (*n* = 5), and appreciated the support received from the coach (*n* = 3). Additionally, caregivers highlighted that after this experience, their child became more aware of their own skills and became more motivated (*n* = 1), or showed better adaptive skills (*n* = 2). Caregivers reported a better understanding of their child's skills and that they gained new tools for the future (*n* = 2). One family expressed the hope that EXPO will become a therapy. Finally, one family expressed the need for a more prolonged period of work with EXPO, and another suggested reducing the number of tests at baseline and post‐intervention.

## 4 Discussion

A primary aim of the present study was to describe the Italian implementation of EXPO, a syndrome‐informed parent‐mediated intervention designed to improve EF in preschool‐aged children with DS. Results demonstrated that households were able to consistently implement the 2.0 version of EXPO across a 12‐week period, with most families able to organise their daily routines to incorporate EXPO activities as much as possible (for in depth analysis of intervention adherence, see Pinks et al. forthcoming). Most activities were led by mothers and primarily conducted at home, often in the afternoons. However, families reported implementation across various times of day, in a range of contexts, and with a variety of participation partners, suggesting that EXPO activities were adaptable from household to household. Some families selected adaptive activities more frequently, and others selected play‐based activities. Furthermore, the reported duration of sessions, mostly within the recommended 5–15 min, confirms that EXPO can potentially fit well into the daily lives of families.

A second aim of the study was to explore the acceptability and enjoyability of EXPO. Data from EXPO activity logs, as well as MP and ET questionnaires, were analysed to gather feedback from caregivers and characterise appraisals of intervention activities and design. Both caregivers and children reported high levels of enjoyment throughout the intervention period, indicating that the activities are well‐designed and appropriate for the target population. This positive trend in engagement and enjoyment is crucial, as positive experiences are critical for maintaining motivation and long‐term adherence to interventions. Families reported appreciation for the perceived effectiveness, enjoyability, variety and ease of integrating the activities into daily life, all of which contributed to the successful implementation of the intervention.

Caregivers reported that they valued the coaching sessions, which provided practical advice on activities and adaptive strategies tailored to their child's specific needs, while also helping to maintain caregivers' motivation. This highlights the importance of ongoing support in caregiver‐mediated interventions. Caregivers also reported increased awareness of their children's abilities, observing improvements not only in EF skills directly targeted by the intervention but also in adaptive behaviours, such as increased engagement, attention, independence, and cooperation in daily activities. Additionally, they perceived that their child demonstrated improvements in problem‐solving, reciprocity, and turn‐taking, as well as more structured individual play by the child and more constructive playtime with caregivers. These parental perceptions suggest that EXPO may not only support cognitive development but also lead to practical outcomes that enhance both adaptation and quality of life. Of course, the efficacy of EXPO must also be demonstrated through a systematic analysis of child performance. However, parental perceptions of the program's effects can contribute to describing its acceptability and enjoyment for families.

Although families expressed overall satisfaction with the program, some challenges were reported. A notable issue was the difficulty some families faced in finding time for activities and incorporating them into daily routines. Some families found the last activities to be too challenging, while others expressed a need for more time to consolidate their child's skills. Additionally, some caregivers struggled with keeping their child engaged or adapting the activities to meet their child's specific needs. These findings suggest that, while EXPO offers valuable opportunities for EF development, further refinement of the activities, particularly to increase accessibility for children with varying motor and cognitive abilities, may improve its future effectiveness.

### 4.1 Limitations

Considering the preliminary nature of this study, a key limitation of this study is its small sample size. For this reason, the results should be interpreted with caution. Future investigations should evaluate EXPO with larger samples across a larger geographic region and in cross‐national contexts. Additionally, since part of the research findings were derived from the EXPO Activity Log, a limitation of this study is that the intervention currently relies on the use of online tools, which may not be broadly accessible to the general population due to varying levels of familiarity and access to technology. A potential way to address this issue could be to provide more intensive support to these families, helping them master the technological aspects involved in EXPO.

A similar issue arises regarding the MP and ET feedback questionnaires. These measures were optional and anonymous to encourage families to freely express their thoughts and share insights they deemed important for improving the EXPO program. However, this approach made it difficult to ensure full participation from all families and to link observations and suggestions to specific children's situations or characteristics. Fewer families completed the feedback questionnaire at the end of the intervention than at midpoint, and those who did not provide optional feedback at endpoint may have held opinions that are different from those who did. Such information could be valuable for enhancing the program and better tailoring it to diverse needs within the DS population.

## 5 Conclusion

The results of this study suggest that EXPO is acceptable to Italian families of young children with DS, and that activities were implemented in a variety of ways across households. Parents also provided several suggestions for further improvements. Based on this feedback, the next steps for the EXPO refinement should involve revising and improving its structure and activities to address the identified limitations and enhance the program in line with the findings of this research. The adjustments should be informed by the data collected during this preliminary implementation trial, with the goal of making the EXPO intervention more adaptable and acceptable on a larger scale. Once this feedback is integrated and the final version of EXPO is completed, a randomised controlled trial will be necessary to assess the program's effectiveness in enhancing EF in preschoolers with DS.

## Author Contributions

Conception and design: Sara Colaianni, Sara Onnivello, Chiara Marcolin, Elisa Rossi, Francesca Pulina, Silvia Lanfranchi and Deborah Fidler. Data analysis and interpretation: Sara Colaianni, Chiara Marcolin, Elisa Rossi, Silvia Lanfranchi and Deborah Fidler. Manuscript drafting: Sara Colaianni, Silvia Lanfranchi and Deborah Fidler. Manuscript review and editing: All authors.

## Ethics Statement

The study protocol and procedures were approved by the Institutional Review Board (IRB) at Colorado State University and the University of Padua Ethics Committee. Caregivers gave informed consent prior to the initiation of any study activities for each participant. The study was performed in accordance with the Declaration of Helsinki.

## Conflicts of Interest

The authors declare no conflicts of interest.

## Data Availability

The data that support the findings of this study are available from the corresponding author upon reasonable request.

## Appendix A Feedback Questionnaire, Mid‐Point. Translated From Italian

### Questions Expo Mid‐Point

Dear Parents,

Thank you for your participation in the EXPO programme.

We ask you for a few minutes of your time to fill out this short mid‐point questionnaire that will help us document and better understand the positive aspects and those that could be improved instead.

The questionnaire is anonymous!

- How old is your child? _______
- What is the gender of your child?
  – Male
  – Female
  – Other
- In this first period, what were the positive aspects of the EXPO programme?
  ___________________________________________________________________________
- Did you find difficulties in carrying out this first part of the EXPO programme? If yes, what were they?
  ___________________________________________________________________________
- Did you prefer game‐based activities or activities based on daily living skills? Why?
  __________________________________________________________________________
- So far, which activities have you enjoyed the most? Why?
  ___________________________________________________________________________
- So far, are there any activities that you have not liked? Which ones and why?
  ___________________________________________________________________________
- Any additional comments you would like to report?
  ___________________________________________________________________________

## Appendix B Feedback Questionnaire, Exit. Translated From Italian

### Questions Expo Exit

Dear Parents,

Thank you for your participation in the whole EXPO program. Congratulations, good job, well done!

We ask you for a few minutes of your time to fill out this short end‐term questionnaire that will help us document and better understand the positive aspects and those that could be improved instead.

The questionnaire is anonymous!

- How old is your child? _______
- What is the gender of your child?
  – Male
  – Female
  – Other
- In this second period, what were the positive aspects of the EXPO program?
  ___________________________________________________________________________
- Did you find difficulties in carrying out this second part of the EXPO program? If yes, what were they?
  ___________________________________________________________________________
- Did you prefer game‐based activities or activities based on daily living skills? Why?
  ___________________________________________________________________________
- Which activities have you enjoyed the most? Why?
  ___________________________________________________________________________
- Are there any activities that you have not liked? Which ones and why?
  ___________________________________________________________________________
- What could be done to improve the EXPO program?
  ___________________________________________________________________________
- How did you find the use of the EXPO App?
  ___________________________________________________________________________
- How did you find the use of the Moodle EXPO site?
  ___________________________________________________________________________
- During these weeks do you think the program has helped your child acquire new skills?
  – Yes
  – No
- If yes, which ones?
  ___________________________________________________________________________
- In your opinion were there any effects on everyday life?
  – Yes
  – No
- If yes, which ones?
  ___________________________________________________________________________
- Have other caregivers (e.g., teachers, babysitters, therapists, grandparents) reported effects to you on everyday life?
  – Yes
  – No
- If yes, which ones?
  ___________________________________________________________________________
- During or at the end of EXPO, did you change anything in the way you approach your child?
  – Yes
  – No
- If yes, what/how?
  ___________________________________________________________________________
- Was it helpful to have weekly coaching sessions?
  – Yes
  – No
- If yes, in what aspects did it facilitate you?
  ___________________________________________________________________________
- Any additional comments you would like to report?
  ___________________________________________________________________________
